# The Impact of Combined Intravenous and Topical Tranexamic Acid in Abdominoplasty with Scarpa Fascia Preservation: A Pilot Study

**DOI:** 10.1007/s00266-026-05977-y

**Published:** 2026-08-12

**Authors:** Carolina Teixeira, Marco Rebelo, António Sousa Barros, António Costa-Ferreira

**Affiliations:** 1 Department of Surgery and Physiology, Faculty of Medicine, Porto University, Porto, Portugal; 2 Department of Plastic Surgery, Portuguese Institute of Oncology, Porto, Portugal; 3 Department of Plastic and Reconstructive Surgery, São João University Hospital, Porto, Portugal

**Keywords:** Abdominoplasty, Tranexamic acid, Plastic surgery, Dual plane abdominoplasty, Scarpa sparing abdominoplasty

## Abstract

**Background:**

Abdominoplasty is a common body contouring procedure. Optimization of surgical outcomes should be pursued. Tranexamic acid (TXA), an antifibrinolytic agent, has demonstrated benefits in various surgical fields, presenting as a potential solution to address these challenges. This study evaluates the impact of combined TXA administration in patients undergoing abdominoplasty with Scarpa fascia preservation.

**Methods:**

A retrospective analysis of 141 patients undergoing full abdominoplasty was performed. Patients were divided into two groups: TXA Group (*n* = 61, receiving 1 g intravenous TXA at anesthesia induction and 50 mL of a 3% TXA solution applied topically before wound closure) and no-TXA Group (*n* = 80, no TXA administration). Postoperative outcomes were compared, including suction drain output, duration of drain use, length of hospital stay, and complication rates (local and systemic).

**Results:**

Groups were homogeneous in baseline characteristics except for age. TXA use resulted in a 27% reduction in total drain output (*p* < 0.001), shorter drain use (*p* < 0.001), and hospital stay (*p* < 0.001), with 81% lower odds of prolonged drain use (OR 0.19, 95% CI [0.06, 0.57]) In the TXA group, drains could be removed on the first day in 42% of the cases as opposed to 2% in the no-TXA group (*p* < 0.001). No statistically significant differences were observed in postoperative complications, including seroma, hematoma, wound healing issues, or thromboembolic events.

**Conclusions:**

Combined TXA use in abdominoplasty with Scarpa fascia preservation significantly reduces drain output, hospital stay, and time until drain removal without increasing complication rates. These findings support TXA’s potential role in improving recovery after abdominoplasty.

**Level of Evidence III:**

This journal requires that authors assign a level of evidence to each article. For a full description of these Evidence-Based Medicine ratings, please refer to the Table of Contents or the online Instructions to Authors www.springer.com/00266.

**Supplementary Information:**

The online version contains supplementary material available at https://doi.org/10.1007/s00266-026-05977-y.

## Introduction

In 2023, abdominoplasty became the 4th most common plastic surgery procedure worldwide, with over 1,2 million surgeries completed, reflecting a 24.8% increase from 2019 [1]. This surge highlights the increasing popularity of body contouring procedures, particularly abdominoplasty.

Despite its aesthetic and functional benefits, abdominoplasty carries several risks and complications [2]. Such issues may prolong recovery, increase hospital stay, and sometimes necessitate additional surgery, impacting patient satisfaction and escalating healthcare costs [3]. Minimizing these risks is essential for optimizing patient outcomes and ensuring overall safety during abdominoplasty.

Tranexamic acid (TXA) is emerging as an effective strategy to mitigate bleeding and reduce inflammation in several surgical procedures [4,5]. As a synthetic antifibrinolytic agent, TXA inhibits plasminogen’s activation to plasmin, thereby preventing plasmin degradation of fibrin clots. Furthermore, it has demonstrated anti-inflammatory properties [6]. Studies have consistently shown that TXA, when used appropriately, is safe, has very few contraindications, and does not increase the risk of thromboembolic events in the general population [7–9]. TXA has well-established efficacy across multiple surgical fields, including trauma, orthopedics, gynecology, and cardiac surgery, but its application in plastic surgery procedures remains relatively underexplored [5, 10–12]. Despite its recently reported usage exceeding 80% in plastic surgery, comprehensive data on its effectiveness in abdominoplasty remain limited [13]. It is essential that what is becoming a routine practice be supported by strong evidence [13].

Both intravenous and topical applications of TXA have been shown to effectively reduce post-surgical complications with standard doses of 10–20 mg/kg for intravenous use and a 3% TXA saline solution for topical application [14,15]. The combined intravenous and topical TXA utilization has been shown to be superior to the intravenous administration alone [16–18]. TXA has a half-life of approximately 2 hours, and approximately 95% is excreted through the kidneys [19].

This study aims to evaluate the impact of combined intravenous and topical TXA use in abdominoplasty, specifically its effects on recovery times and the incidence of postoperative complications.

## Materials and Methods

This study was designed as a retrospective observational cohort study, focusing on patients who underwent full abdominoplasty with Scarpa fascia preservation in Santa Casa Misericórdia Lousada Hospital between March 2023 and December 2024 with findings intended to inform the design of future randomized controlled trials. The Ethical Committee of this institution approved the current study. All patients enrolled in this study signed informed consent documents.

### Patient Selection

The selected patients had abdominal deformities characterized by excess skin, adipose tissue, and/or muscle laxity and met the criteria for abdominoplasty. [20] Exclusion criteria included significantly elevated operative health risks, a body mass index (BMI) over 30 kg/m^2^ (except for patients with previous bariatric surgery), and plans for future pregnancy. Smokers were asked to suspend smoking 4 weeks before surgery.

A total of 141 patients were included in the study and classified into two groups: no-TXA and TXA. In the former, no TXA was used. In the latter, TXA was used in combined therapy.

The same surgical team performed all the procedures using the same surgical technique except for incorporating combined TXA therapy during the study period.

### Surgical Technique

All patients underwent an abdominoplasty with Scarpa fascia preservation, umbilical transposition, plication of the rectus abdominis muscle, and flank liposuction, following the method described by Costa-Ferreira. [21,22]

Preoperative enoxaparin(40 mg/d subcutaneously during the hospital stay, starting at least two hours before surgery)and broad-spectrum intravenous antibiotics were routinely administered. For patients receiving TXA, a preoperative intravenous dose of 1 g was administered during induction. The surgical procedure began with prepping and draping the patient under general anesthesia. Dissection was done with electrosurgery (ERBE VIO 300 S–Erbe Elektromedizin GmbH, Tuebingen, Germany)in the coagulation mode with the same settings in all surgeries: coagulation set to forced coagulation, effect 4, maximum 50 Watts (spray mode deactivated). A limited flank liposuction was performed, but the upper abdominal flap was not liposuctioned. No progressive tension (quilting) sutures were used. Two or three closed-suction drains were applied with exit points on the mons pubis. For patients receiving TXA, a topical application of 50 mL of a 3% TXA solution was done just before wound closure, corresponding to 1500 mg of TXA. Half that volume was applied to the full width of the surgical field, and the wounds were temporarily closed with staples. The rest of the TXA solution was applied through the umbilical incision on the epigastric area and the lower abdominal incision in the infraumbilical area. The suction drains were connected after the final bandage was applied. Compression garments were applied in the operating room before the patient awoke.

Drains were removed when the patient was ambulatory, and the output per day was ≤30 mL collected over 24 hours on each drain or when the drain output was lower than 10 mL on two consecutive evaluations.

Patients were observed by the surgeon during the hospital stay, once per week for 1 month, and then every 6 months until full scar maturation was achieved. Additional medical appointments were scheduled according to each patient’s clinical evolution.

### Data Collection

Data were retrospectively collected from the patient's medical records and included: age, gender, body mass index (BMI) before abdominoplasty, smoking habits, medical comorbidities, previous abdominal surgical procedures, and surgical specimen weight.

The outcomes investigated were drain output(registered at 8 am, 1 pm, and 8 pm by a nurse), duration of suction drain use and hospital stay, emergency department visits(related to surgery), readmission to the hospital, reoperation, and complications. Systemic complications comprised blood transfusion, thromboembolic events(deep vein thrombosis/pulmonary embolism), and death. Local complications included seroma, hematoma/bleeding, wound infection, healing issues(wound dehiscence without necrosis), and skin necrosis.

### Data Analysis

The analytical approach comprised three components, all conducted in R programming language, version 4.5.0 [23]. First, descriptive statistics characterized the study population through means, standard deviations, and ranges for continuous variables, and frequencies with percentages for categorical variables, encompassing patient demographics, clinical characteristics, and surgical details. Second, the primary analysis employed ordinal logistic regression to model drain duration as an ordered categorical outcome. This method was specifically chosen to preserve the ranked nature of the drain duration categories while examining the influence of clinically relevant predictors, including patient factors (age, BMI, smoking status, and previous abdominal surgery), surgical variables (surgeon, operative specimen weight), and postoperative characteristics (treatment allocation, total drain volume). Variable selection was guided by clinical expertise and surgical experience regarding factors most likely to affect postoperative drain duration. Results are expressed as adjusted odds ratios with 95% confidence intervals, indicating the likelihood of extended drain placement. Finally, longitudinal visualization tracked daily drain output patterns across all patients to identify temporal trends and characterize drain volume trajectories throughout the postoperative period.

## Results

A total of 141 patients were included in the study(no-TXA group, 80 patients; TXA group, 61 patients). Table 1 summarizes patient characteristics. Most patients were female. The groups were homogeneous except for age, with higher values in the TXA group. The two surgeons were equally represented in both groups

**Table 1:** General demographic and clinical characteristics, stratified by group (N = 141). [no-TXA Group: Abdominoplasty without TXA (N = 80); TXA Group: Abdominoplasty using TXA (N = 61)].

Characteristic	no-TXA (*N* = 80)	TXA (*N* = 61)	*p*-value
Surgeon M.R., *n* (%)	40 (50%)	29 (48%)	0.8
Age			**0.017**
Median (IQR)	41 (36, 47)	45 (39, 50)	
Range	[21, 61]	[25, 64]	
BMI before abdominoplasty (kg/m^2^)			0.2
Median (IQR)	26.0 (23.9, 27.6)	26.6 (24.2, 27.8)	
Range	[18.7, 31.0]	[20.8, 32.9]	
Previous abdominal surgeries, n (%)	45 (56%)	38 (62%)	0.5
Smoker, *n* (%)	7 (8.8%)	7 (11%)	0.6
Operative specimen weight (g)			0.6
Mean ± SD	898.0 ± 363.0	994.0 ± 464.0	
Range	[270.0, 1,972.0]	[304.0, 2,300.0]	

Statistically significant differences between groups in a certain characteristic or outcome is given in bold p-values

*Range*, [minimum, maximum]*; BMI*, body mass index; *SD*, standard deviation

Pearson’s Chi-squared test, Wilcoxon rank sum test, and Fisher’s exact test were used

Outcomes are presented in Table 2 with multivariable adjusted analyses provided in Supplementary Table S1. The TXA group presented significantly shorter hospital stays and shorter time until drain removal. In the TXA group, 41% of patients were discharged after one day and 49% after two days, compared to 2.5% and 88% in the no-TXA group (*p* < 0.001). Similarly, 43% of TXA patients required a drain for only one day and 48% for two days, whereas 88% of the no-TXA group required at least two days, and as little as 2.5% used drains for one day (*p* < 0.001).

**Table 2:** Outcomes of both groups (*N* = 141). [no-TXA Group: Abdominoplasty without TXA (*N* = 80); TXA Group: Abdominoplasty using TXA (*N* = 61)]

Characteristic	no-TXA (*N* = 80)	TXA (*N* = 61)	*p*-value
Hospital stay, *n* (%)			**< 0.001**
Day 1	2 (2.5%)	25 (41%)	
Day 2	70 (88%)	30 (49%)	
Day 3	7 (8.8%)	6 (9.8%)	
Day 4	1 (1.3%)	0 (0%)	
Time until drain removal, *n* (%)			**< 0.001**
Day 1	2 (2.5%)	26 (43%)	
Day 2	70 (88%)	29 (48%)	
Day 3	7 (8.8%)	6 (9.8%)	
Day 4	1 (1.3%)	0 (0%)	
Total drain output (mL)			**< 0.001**
Mean ± SD	186.0 ± 91.0	135.0 ± 110.0	
Range	[40.0, 460.0]	[0.0, 560.0]	
Total day 1 drain output (mL)			**0.010**
Mean ± SD	137.0 ± 73.0	104.0 ± 72.0	
Range	[0.0, 310.0]	[0.0, 290.0]	
Total day 2 drain output (mL)			0.3
Mean ± SD	46.0 ± 33.0	47.0 ± 52.0	
Range	[0.0, 140.0]	[0.0, 180.0]	
Total day 3 drain output (mL)			0.3
Mean ± SD	41.0 ± 34.0	48.0 ± 82.0	
Range	[15.0, 120.0]	[0.0, 170.0]	
Total day 4 drain output (mL)	60.0*	-	
Local complications, *n* (%)			
Liquid collections			
Seroma	6 (7.5%)	9 (15%)	0.2
Hematoma	2 (2.5%)	2 (3.3%)	> 0.9
Healing problems	12 (15%)	4 (6.6%)	0.12
Skin necrosis	1 (1.3%)	0 (0%)	> 0.9
TVP/TEP	0 (0%)	0 (0%)	–
Readmission, *n* (%)	2 (2.5%)	0 (0%)	0.5
Reoperation, *n* (%)	2 (2.5%)	0 (0%)	0.5

Statistically significant differences between groups in a certain characteristic or outcome is given in bold p-values

* Range*, [minimum, maximum]*; SD*, standard deviation

Pearson’s Chi-squared test, Wilcoxon rank sum test, and Fisher’s exact test were used

*Only one patient presented drainage values on this day

Total drain volume was significantly lower in the TXA group, with the largest difference observed on the first postoperative day. On subsequent days, differences in drain output were not statistically significant. The macroscopic appearance of the suction drain content in the TXA group was consistently clearer than in the no-TXA group, suggesting a major decrease in the hematic content.

**Fig. 1:**
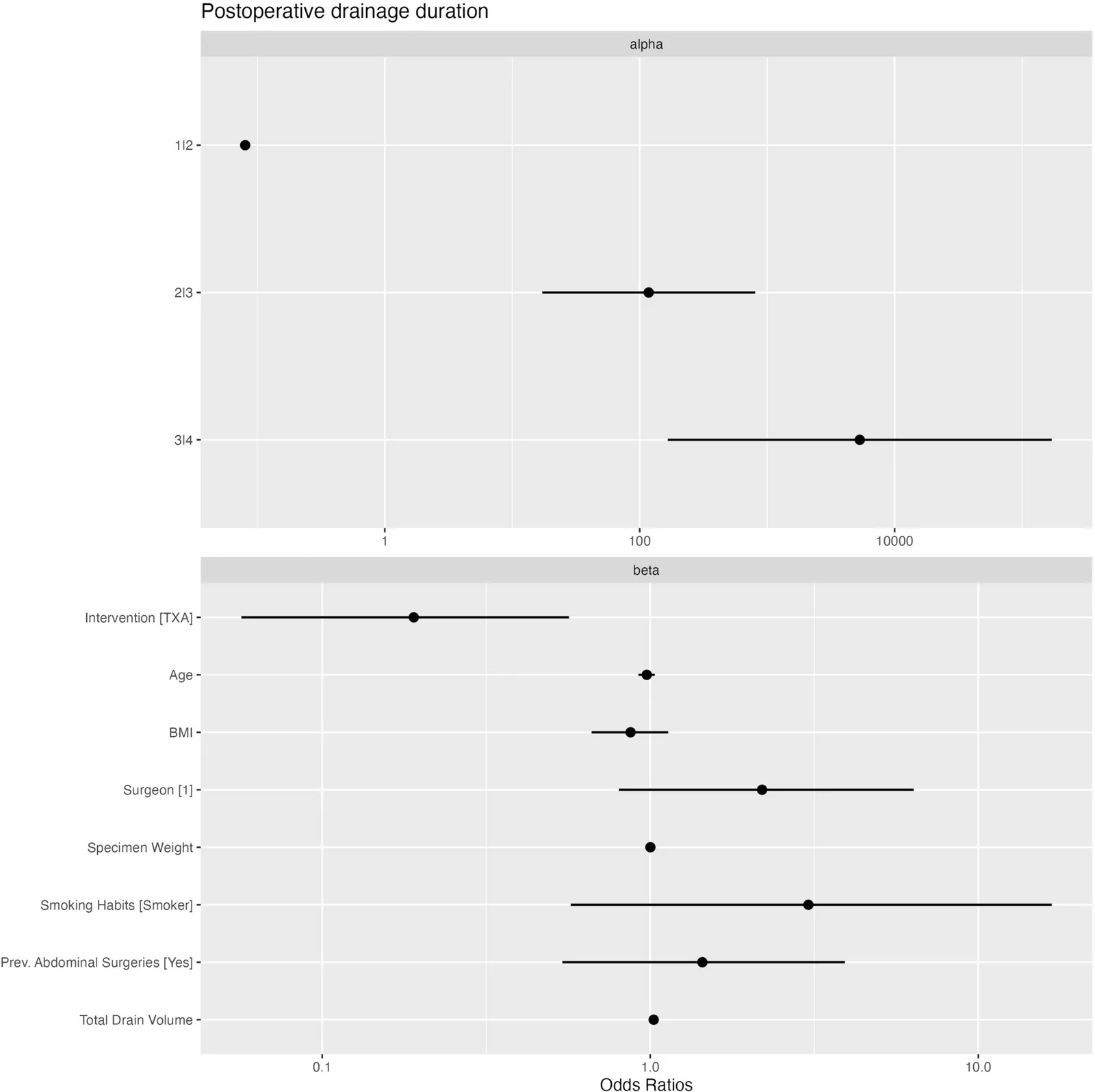
Forest plot illustrating the results of ordinal logistic regression analysis for factors associated with surgical drain duration. The upper panel ("alpha") represents the threshold parameters between drain duration categories (1|2, 2|3, and 3|4). The lower panel ("beta") presents the odds ratios with 95% confidence intervals for various clinical predictors, including treatment with TXA, total drain output, and patient demographics. Points to the left of the reference line (OR=1) indicate negative associations, and points to the right indicate positive associations. Confidence intervals that do not cross the reference line signify statistically significant effects

Ordinal logistic regression analysis (Fig. 1, Table 3) further supported that TXA administration significantly reduced the odds of prolonged drain use (OR 0.19, 95% CI [0.06, 0.57 ]), corresponding to an 81% reduction in the odds of a longer drain duration. Total drain volume was the other significant predictor (*p* < 0.001), with each unit increase associated with 2% higher odds of extended drainage (OR 1.02, 95% CI [1.02, 1.03]). Other patient-related factors, such as age, BMI, smoking habits, previous abdominal surgeries, and specimen weight, were not significantly associated with drain duration.

**Table 3:** Multivariable ordinal logistic regression analysis of factors associated with postoperative drainage duration

Characteristic	OR	95% CI	*p*-value
Intervention			0.002
No-TXA	–	–	
TXA	0.19	0.06, 0.57	
Age	0.98	0.92, 1.03	0.4
BMI	0.87	0.66, 1.13	0.3
Surgeon			0.13
0	–	–	
1	2.19	0.80, 6.35	
Specimen weight	1.00	1.00, 1.00	0.2
Smoking habits			0.2
No smoker	–	–	
Smoker	3.03	0.57, 16.7	
Prev. Abdominal surgeries			0.5
No	–	–	
Yes	1.44	0.54, 3.92	
Total drain volume	1.02	1.02, 1.03	< 0.001

*CI*, confidence interval; *OR*, odds ratio

**Fig. 2:**
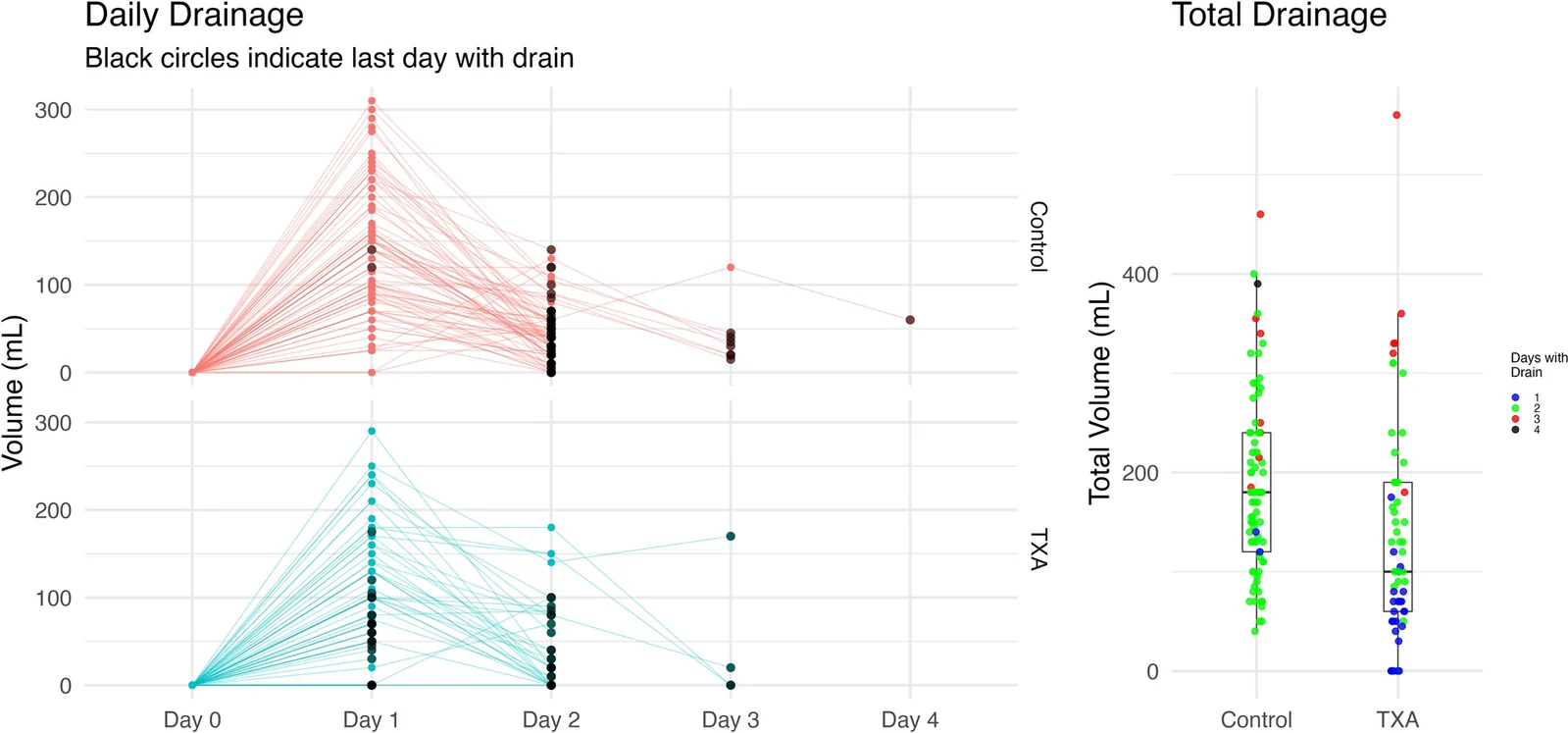
Comparison of drainage volume between the control and TXA groups. The left panel illustrates daily drainage volume (mL) over the first four days post-surgery for individual patients. Black dots indicate the last day the drain was in place for each patient. Red lines represent the control group, while blue lines represent the TXA group. The right panel presents a box-whisker plot of total drainage volume (mL) over the entire drainage period. The boxes represent the interquartile range (IQR), with the lower and upper boundaries indicating the 25th and 75th percentiles, respectively. The solid horizontal line within each box marks the median volume. Whiskers extend to the smallest and largest values within 1.5 times the IQR from the box boundaries. Individual colored dots correspond to patients, grouped by the number of days the drain remained in place: blue (1 day), green (2 days), yellow (3 days), and red (4 days)

Analysis of drainage patterns (Fig. 2) revealed a consistent downward trend in both groups, with the TXA group consistently demonstrating lower median drainage volumes. The no-TXA group exhibited greater variability, with several outliers exceeding 300 mL, whereas the TXA group had more uniform and lower drainage output.

Table S1 presents the analysis that examines factors influencing postoperative outcomes comparing TXA versus no-TXA across four key complications: seroma, hematoma, wound complications, and hospital stay. The most relevant finding was hospital length of stay, where TXA application demonstrated a significant effect with an OR of 0.23 (95% CI 0.07–0.66, *p* = 0.006), representing a 77% reduction in the odds of prolonged hospitalization. This benefit occurred without any increase in surgical complications. Indeed, TXA showed no statistically significant association with seroma formation, hematoma, or wound complications, suggesting its safety profile. Total drain volume was another key factor, significantly predicting both increased seroma formation (OR 1.01, *p* = 0.039) and longer hospital stays (OR 1.02, *p* < 0.001), with each unit increase in drainage associated with worse outcomes. Traditional patient risk factors—age, BMI, smoking history, previous abdominal surgeries, specimen weight, and surgeon variability—showed no significant associations with any of these outcomes in the adjusted analysis.

Rates of postoperative complications (Table 2) were similar between groups. Seroma formation was more frequent in the TXA group than in the no-TXA group, though this difference was not statistically significant. Hematoma rates were comparable, as were rates of skin necrosis. Wound healing complications were slightly higher in the no-TXA group, but this difference did not reach statistical significance. No thromboembolic events, blood transfusions, or deaths were observed in either group.

## Discussion

Despite the widespread use of tranexamic acid (TXA) in various surgical fields, including plastic surgery, its role in abdominoplasty remains underexplored [5, 10–13]. Our findings provide evidence that the administration of combined TXA therapy (intravenous plus topical) in patients undergoing abdominoplasty with Scarpa fascia preservation is associated with a significant reduction in postoperative drain output, decreased time to drain removal, and hospital stay. Total drain volumes in the TXA group were reduced by 27.4%, with the effect more pronounced on the first postoperative day.

These results are biologically plausible and can be explained, given TXA’s antifibrinolytic mechanism. TXA is a synthetic analog of lysine that inhibits the conversion of plasminogen to plasmin by blocking the lysine-binding sites of plasminogen. This prevents fibrin degradation and stabilizes blood clots, consequently minimizing residual bleeding [24]. Furthermore, plasmin inhibition reduces plasmin-induced platelet activation, preserving more circulating platelets to support clot formation [24]. TXA also prevents premature clot breakdown, promoting effective coagulation and reducing the reliance on prolonged drainage [24]. Another mechanism through which TXA might reduce the drain output is by addressing the inflammatory response. In abdominoplasty, extensive tissue dissection triggers inflammation, increasing exudative serous fluid production [25,26]. TXA has been shown to lower markers of inflammation, such as interleukin 6 and creatine kinase, thereby reducing inflammation and, subsequently, serous fluid production [6]. These effects may explain the consistently lower drain volumes that resulted in earlier drain removal and shortening of hospital stay. On the other hand, they may also explain the reported recurrent macroscopic non-hematic appearance of the suction drain fluid.

Many strategies have been investigated to address abdominoplasty complications and enhance surgical outcomes [1]. Closed suction drains remain a frequent approach for seroma prevention, the most prevalent complication of the procedure [27–29]. In the most comprehensive survey on abdominoplasty, Matarasso reported that 98% of the surveyed surgeons utilized suction drains in their procedures [30].

Scarpa fascia preservation has demonstrated favorable outcomes in reducing drain output and duration of suction drain use after an abdominoplasty [31,32]. A randomized controlled trial also reported a very good aesthetic result, faster sensibility recovery, and high scar quality [31, 33]. Similarly, Pisco observed slightly improved results with the same approach, including shorter drain removal times and lower drain output [34]. Similar results were reported in bariatric patients with an important reduction of long drainers [35–37]. A recently published systematic review concluded “that the preservation of Scarpa fascia during an abdominoplasty procedure should be considered as a routine practice, because it has been shown to result in reduced seroma incidence rates and faster drain removal” [38]. A recent survey of members of the American Society of Plastic Surgery found that more than 22% used this technique [39]. The previous studies reported higher values of drain output and time to drain removal than those observed in our research, suggesting that adding TXA may further enhance these outcomes, achieving periods of suction drain use in the 24–48 h range after a full abdominoplasty.

TXA has been extensively studied in several surgical fields, consistently reducing blood loss and complications without increasing thromboembolic risks [9–11, 40]. In plastic surgery, the increasing number of studies exploring the use of TXA signals a growing trend toward expanding its application within the specialty [41]. In breast surgery, Ausen demonstrated that median fluid output decreased by 42% (*p* = 0.017) in patients receiving topical TXA during reduction mammoplasty [42]. However, evidence regarding TXA remains limited and somewhat contradictory in abdominoplasty procedures. Watchel reported that 1 g intravenous TXA in Fleur-de-lis abdominoplasty or apronectomy significantly reduced total drain output by 42.5% (*p* = 0.0017) and shortened the hospital stay [43]. Watchel’s study used a classical dissection plane on top of the deep fascia. Zaussinger reported a statistically significant reduction in seroma formation and the need for postoperative seroma aspiration with intravenous TXA after abdominoplasty. However, no statistically significant differences were found in the median drain output [44]. The latter study used a bodyweight-adapted dosage of TXA (1-2 g) administered intravenously after specimen resection before wound closure. The abdominoplasty technique in Zaussinger’s study used a dissection plane on the thickness of the deep fat, a similar principle to the Scarpa sparing technique. Bayter-Marin published a randomized controlled trial demonstrating that TXA improves recovery after abdominoplasty when used preoperatively and continued for 48 hours after surgery [45].

Conversely, other studies have reported no significant benefits associated with TXA use in abdominoplasty. Habarth-Morales reviewed a multicenter federated electronic health record database of patients submitted to breast reduction or abdominoplasty [46]. They reported no differences in hematoma or seroma rates with intravenous TXA [46]. There is no information regarding the clinical profile of the study population, the type of abdominoplasty procedure, or the dose and timing of TXA treatment. Weissler investigated isolated topical TXA in panniculectomy procedures and reported no reduction in hematoma, seroma, or drain duration [47]. The latter study used 3 g of TXA in 75 mL of NaCl 0.9% delivered topically (3% TXA solution) directly to the wound surfaces and indirectly through the suction drains retrogradely.

These conflicting findings highlight the need for further high-quality studies to clarify TXA’s role in abdominoplasty. Differences in study design, TXA administration methods (intravenous vs. topical), patient populations, and specific abdominoplasty surgical techniques may contribute to the variability in outcomes.

One of the primary concerns regarding TXA use is the potential increased risk of thromboembolic complications. This assumes particular relevance as abdominoplasty is the aesthetic procedure most associated with DVT/PTE [48]. The underlying mechanism is still unclear. This concern may prevent the use of TXA in abdominoplasty surgery, but the current reality seems to be very different. A recent survey of the British Association of Aesthetic Plastic Surgeons members reported that 78% of the surveyed surgeons use TXA and that abdominoplasty is the procedure with the highest use of TXA, peaking at 84 % of utilization [13]. Considering these findings, it is urgent to gather evidence on the use of TXA in abdominoplasty procedures.

Our study found no thrombotic events, consistent with extensive data that concluded that TXA does not increase the risk of thromboembolic events when used within recommended doses [5, 8, 49–51]. In addition, this study’s results (Table S1) provide evidence for TXA's clinical utility. The 77% reduction in odds of prolonged hospitalization, coupled with the absence of increased complication rates across all measured outcomes, suggests that TXA accelerates recovery and reduces healthcare utilization without compromising surgical safety, making it both clinically beneficial and economically advantageous. The lack of association between TXA and direct surgical complications, including seroma, hematoma, and wound issues, provides robust evidence for its routine use in this surgical context.

The rationale for combined TXA therapy lies in optimizing its antifibrinolytic and anti-inflammatory effects while maintaining safety. Robust evidence from other surgical disciplines indicates that a dual-administration strategy, both systemic and local, is effective and safe for optimizing hemostatic outcomes while mitigating the risks associated with high systemic exposure [16–18, 52]. The combined use of intravenous and locally applied TXA for abdominoplasty has not been comprehensively studied. TXA intravenous administration of 1 g during induction has been shown to block up to 80% of fibrinolysis [53]. The studies by Zaussinger and Wachtel have demonstrated efficacy in intravenous TXA use in abdominoplasty procedures [43,44]. However, Weissler’s findings suggest that topical TXA alone does not offer benefits in this procedure [47]. Topical TXA has the advantage of low systemic absorption, as highlighted by Ausen, who reported serum concentrations unlikely to cause systemic effects [54]. This important conclusion was based on a pharmacokinetic study of TXA after topical application in patients submitted to abdominoplasty after massive weight loss [54].

This is the first study to investigate the combined use of topical and intravenous TXA in abdominoplasty. Most patients in this study were women, which may affect the applicability of the findings to male patients, who could experience different outcomes. The main limitation of the current study is its retrospective and non-randomized design, which introduces potential selection bias and residual confounding despite statistical adjustments. The lack of data on asymptomatic thromboembolic events limits a comprehensive evaluation of TXA’s safety.

Abdominoplasty can be performed in both inpatient and outpatient settings, but in either case, reducing the duration of drain use is advantageous, as prolonged drainage is linked to increased patient discomfort, a higher risk of infection, and potential delays in recovery [27]. In inpatient procedures, the reduction in hospital stays not only improves patient outcomes but also improves healthcare efficiency and contributes to cost reduction. [3]

## Conclusion

The findings of this pilot study suggest promising potential benefits of combined TXA therapy in abdominoplasty with Scarpa fascia preservation procedures. Patients receiving TXA experienced significantly shorter hospital stays, earlier drain removal, and reduced drainage volumes compared to the control group, despite being older on average. The results confirmed TXA's significant association with reduced drain duration, with TXA patients having 81 % lower odds of requiring prolonged drainage. These advantages could translate to meaningful clinical and economic benefits through faster recovery, reduced care requirements, and more efficient resource utilization.

## Supplementary Information

Below is the link to the electronic supplementary material.

Supplementary file1 (DOCX 21 kb)
