# Supplementary material for: The Impact of Combined Intravenous and Topical Tranexamic Acid in Abdominoplasty with Scarpa Fascia Preservation: A Pilot Study
Source: Aesthetic Plast Surg. 2026 Aug 12;50(20):8260–9. doi: 10.1007/s00266-026-05977-y (PMC13727876; doi:10.1007/s00266-026-05977-y)
Supplement: Supplementary file 1 [file 266_2026_5977_MOESM1_ESM.docx]

**Table S1.** Multivariable logistic and ordinal regression analysis of postoperative outcomes.

|  | Seroma | | | Hematoma | | | Wound complications | | | Hospital stay | | |
| --- | --- | --- | --- | --- | --- | --- | --- | --- | --- | --- | --- | --- |
| **Characteristic** | **OR** | **95% CI** | **p-value** | **OR** | **95% CI** | **p-value** | **OR** | **95% CI** | **p-value** | **OR** | **95% CI** | **p-value** |
| Intervention |  |  | 0.8 |  |  | 0.7 |  |  | 0.5 |  |  | 0.006 |
| No-TXA | — | — |  | — | — |  | — | — |  | — | — |  |
| TXA | 1.24 | 0.26, 5.57 |  | 0.59 | 0.04, 7.13 |  | 0.62 | 0.15, 2.17 |  | 0.23 | 0.07, 0.66 |  |
| Age | 1.08 | 0.99, 1.19 | 0.068 | 1.03 | 0.91, 1.18 | 0.6 | 0.97 | 0.90, 1.03 | 0.3 | 0.97 | 0.92, 1.03 | 0.4 |
| BMI | 1.33 | 0.93, 1.99 | 0.13 | 1.90 | 0.93, 5.04 | 0.080 | 0.90 | 0.68, 1.17 | 0.4 | 0.85 |  | 0.2 |
| Surgeon |  |  | 0.2 |  |  | 0.4 |  |  | 0.8 |  |  | 0.2 |
| 0 | — | — |  | — | — |  | — | — |  | — | — |  |
| 1 | 0.40 | 0.08, 1.79 |  | 0.30 | 0.01, 4.49 |  | 1.17 | 0.39, 3.52 |  | 2.00 | 0.75, 5.65 |  |
| Specimen weight | 1.00 | 1.00, 1.00 | 0.8 | 1.00 | 0.99, 1.00 | 0.11 | 1.00 | 1.00, 1.00 | 0.5 | 1.00 | 1.00, 1.00 | 0.3 |
| Smoking habits |  |  | 0.8 |  |  | 0.2 |  |  | 0.2 |  |  | 0.2 |
| No smoker | — | — |  | — | — |  | — | — |  | — | — |  |
| Smoker | 0.73 | 0.03, 5.96 |  | 0.00 |  |  | 2.76 | 0.54, 11.7 |  | 2.67 | 0.52, 14.1 |  |
| Prev. Abdominal Surgeries |  |  | 0.4 |  |  | 0.8 |  |  | 0.5 |  |  | 0.7 |
| No | — | — |  | — | — |  | — | — |  | — | — |  |
| Yes | 1.97 | 0.47, 10.6 |  | 0.78 | 0.08, 8.07 |  | 1.50 | 0.51, 4.83 |  | 1.25 | 0.47, 3.29 |  |
| Total Drain Volume | 1.01 | 1.00, 1.01 | 0.039 | 0.98 | 0.96, 1.00 | 0.055 | 1.00 | 1.00, 1.01 | 0.3 | 1.02 | 1.02, 1.03 | <0.001 |
| Abbreviations: CI = Confidence Interval, OR = Odds Ratio | | | | | | | | | | | | |
